# Landscape Characteristics and Distribution of Suitable Habitats for the Black-Tailed Godwit During the Non-Breeding Season: A Case Study of the Middle and Lower Yangtze River Region

**DOI:** 10.3390/ani16111592

**Published:** 2026-05-23

**Authors:** Zeng Jiang, Mingqin Shao

**Affiliations:** Key Laboratory of Biodiversity Conservation and Bioresource Utilization of Jiangxi Province, College of Life Sciences, Jiangxi Normal University, Nanchang 330022, China; jiangzeng@jxnu.edu.cn

**Keywords:** Black-tailed Godwit, landscape index, middle and lower Yangtze River, maxent model, correlation analysis

## Abstract

This study examines the landscape characteristics of high-suitability habitats for the Black-tailed Godwit (*Limosa limosa*) during the non-breeding season in inland and coastal wetlands of the middle and lower Yangtze River regions, and seeks to elucidate the distribution patterns and their drivers. Using the MaxEnt model and landscape analysis, the following conclusions were obtained: (1) High-suitability habitats for the Black-tailed Godwit cover approximately 128,800 km^2^ and are primarily distributed across the middle and lower Yangtze River regions. (2) The dominant environmental variables were identified as elevation, distance to water source, slope, distance to paddy field, land use classification, and minimum temperature of the coldest month. (3) Landscape fragmentation, habitat connectivity, human disturbance, and climate change were found to be associated with the shift in the Black-tailed Godwit’s distribution from coastal to inland areas. (4) The distribution of the Black-tailed Godwit in the Nanji Wetland showed significant moderate positive correlation with shallow-water area (*r* = 0.38, *p* < 0.05) and significant moderate negative correlation with deep-water area (*r* = −0.48, *p* < 0.01). (5) At large spatial scales (coastal and inland wetlands), habitat connectivity and fragmentation were found to exert a greater influence, whereas at smaller spatial scales (Nanji Wetland) land use areas (wetlands and shallow-water areas) and food resources were found to exert greater influence on the Black-tailed Godwit’s distribution. This study synthesizes findings from multiple sources and aims to provide a reference for the conservation of the Black-tailed Godwit.

## Simple Summary

Through landscape analysis and modeling, this study revealed that high-suitability habitats for the Black-tailed Godwit (*Limosa limosa*) during the non-breeding season cover approximately 128,800 km^2^. These habitats are mainly located in the middle and lower reaches of the Yangtze River and along coastal wetlands. The dominant environmental variables identified were elevation, distance to water source, slope, distance to paddy field, land use classification, and minimum temperature of the coldest quarter. Landscape fragmentation, habitat connectivity, human disturbance, and climate change were found to be associated with the species’ shift in distribution from coastal to inland habitats. The distribution of the Black-tailed Godwit was primarily influenced by connectivity and fragmentation at large spatial scales (coastal and inland), whereas at smaller scales (the Nanji Wetland), local factors such as shallow-water areas and food resources exerted greater influence.

## 1. Introduction

The maximum entropy model (MaxEnt) is a species distribution model that combines existing species distribution data with environmental variables to predict the potential habitat distribution patterns and dominant environmental factors of a target species [1,2]. This can provide scientific guidance for the conservation or management of that species [1,2]. Currently, this model is widely applied in wildlife research [1,3,4,5,6,7]. Landscape indices such as number of patches (NP), patch density (PD), Maximum Patch Index, etc., are quantitative measures of landscape pattern and serve as important indicators for assessing habitat quality [8,9,10]. Wetland landscape pattern refers to the spatial arrangement and combination of wetland patches, while wetland landscape indices quantify these landscape pattern characteristics, and help researchers to accurately assess the spatial configuration and ecological functions of wetland landscapes [8,10,11,12]. Landscape pattern exerts a significant influence on the behavioral activities and habitat selection of waterbirds [8,13]. Therefore, relevant indices of wetland landscape pattern can accurately assess the health status of waterbird habitats, facilitating the investigation of the relationship between waterbird distribution preferences and landscape pattern. The middle and lower reaches of the Yangtze River are primarily composed of six plains, represented by the Dongting Lake Plain, Poyang Lake Plain, and Yangtze River Delta Plain [14,15]. With a low average elevation, these regions support numerous inland lake wetlands and coastal wetlands [14,15]. These wetlands serve as stopover and wintering sites for many migratory birds, and the spatial configuration of their wetland landscape patterns has a significant impact on the habitation of numerous waterbirds [16,17,18]. Therefore, investigating the correlation between the landscape patterns of inland lakes and coastal wetlands in the middle and lower reaches of the Yangtze River and the distribution of waterbirds can provide a scientific basis for the conservation of waterbirds and their habitats.

The Black-tailed Godwit (*Limosa limosa*) is a medium-sized wader belonging to the family Scolopacidae within the order Charadriiformes [19,20,21]. Most individuals overwinter or migrate through China, while a small number breed in northern regions such as Inner Mongolia and Xinjiang [19,20]. Outside the breeding season, the Black-tailed Godwit has a very wide distribution in China, showing a particular preference for inhabiting and foraging in shallow waters of wetlands such as marshes, lakes, and coastal areas, where it primarily feeds on insects, crustaceans, and mollusks [19,20,21,22]. The Black-tailed Godwit has high environmental requirements and is highly sensitive to environmental changes [19,21]. The Black-tailed Godwit was classified as Least Concern (LC) by the International Union for Conservation of Nature (IUCN) in 2004 [19,21]. In recent years, due to the impact of human activities [23] and global climate change [7,24,25,26], the area of natural habitats for migratory birds such as shorebirds in China has declined, and habitat fragmentation has intensified. In particular, intensified human activities in coastal wetlands such as the Bohai Bay [27], Yancheng Wetlands [28], and Nanhui Dongtan [23] have caused significant destruction of the Black-tailed Godwit’s natural habitats. Consequently, the species has gradually shown a greater preference for inland lake wetlands during the non-breeding season. This human activity has contributed to increased habitat fragmentation for the Black-tailed Godwit in Western Europe, where populations are also undergoing rapid declines [29]. The Black-tailed Godwit was listed as Near Threatened (NT) by the IUCN in 2016 [30].

Current research on the Black-tailed Godwit remains insufficient in China, focusing primarily on its breeding and wintering periods, as well as population sizes in certain regions, migration patterns, and diurnal behavioral rhythms [19,21,22]. The dominant environmental variables and underlying mechanisms governing its distribution during the non-breeding season have not yet been clearly identified [19,21,22]. Previous international studies have primarily examined habitat selection and conservation during the breeding season [29,31], foraging strategies [32,33], migration ecology [34], and the effects of agricultural landscape changes [31]. However, these studies have not yet investigated the relationships between distributional changes and environmental alterations using landscape indices, including patch fragmentation. Furthermore, ecological remote sensing methods provide clear advantages in assessing wetland ecosystem stability [35]. Therefore, this study aims to use the MaxEnt model to identify the potential suitable habitats for the Black-tailed Godwit during its non-breeding season in China and to identify the dominant environmental variables influencing its distribution, and employs supervised classification using satellite remote sensing imagery to quantify landscape indices for coastal wetlands and inland lake wetlands. Finally, distribution data from field biodiversity surveys were used to reveal relationship between Black-tailed Godwit distribution and landscape indices, aiming to elucidate the underlying causes of recent distribution differences between inland lakes and coastal wetlands. By integrating multi-scale findings from the MaxEnt model and landscape indices analysis, this study proposes systematic conservation recommendations, providing foundational reference data for migratory bird conservation planning.

## 2. Study Area

### 2.1. The Middle and Lower Reaches of the Yangtze River

**Figure 1:**
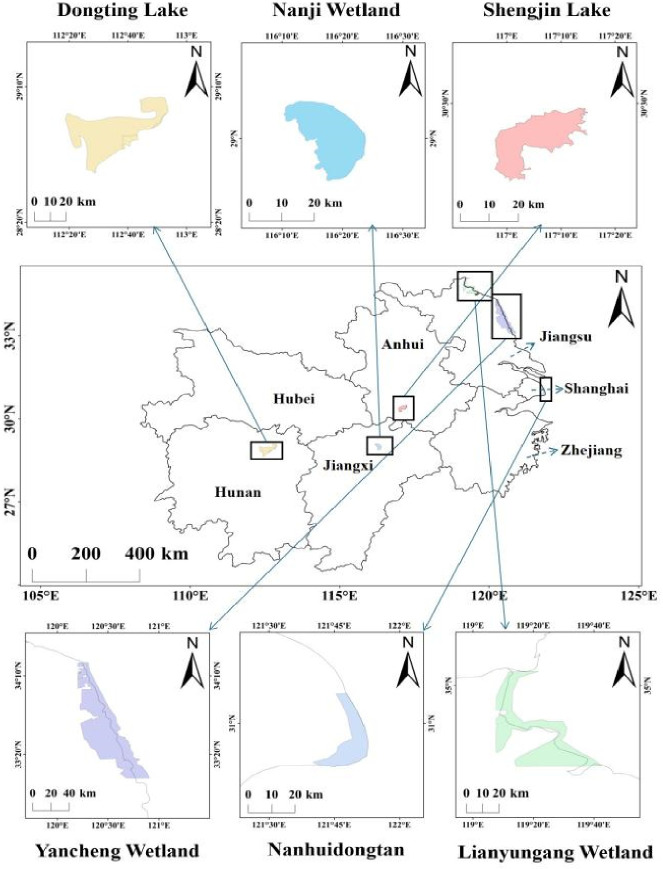
Selected inland and coastal wetlands in the middle and lower reaches of the Yangtze River.

The middle and lower reaches of the Yangtze River include Hunan province, Hubei province, Jiangxi province, Anhui province, Jiangsu province, and Shanghai city, with a total area of approximately 80,000 km^2^ [14,15]. Most of this region lies at an elevation of 5–100 m.a.s.l. and the lowest temperature during the non-breeding season typically hover around −5 °C but can drop as low as −10 °C to −15 °C in some extreme years [36]. The inland lakes in the middle and lower reaches of the Yangtze River selected for this study are key stopover or wintering sites for migratory birds. These include the Nanji Wetland National Nature Reserve in Jiangxi, the Shengjin Lake National Nature Reserve in Anhui, and the Dongting Lake National Nature Reserve in Hunan. The selected coastal wetlands are Nanhuidongtan in Shanghai; the Yancheng Wetland in Jiangsu; and the Lianyungang Wetland in Jiangsu (Figure 1).

### 2.2. Seven Sub-Lakes of the Nanji Wetland

**Figure 2:**
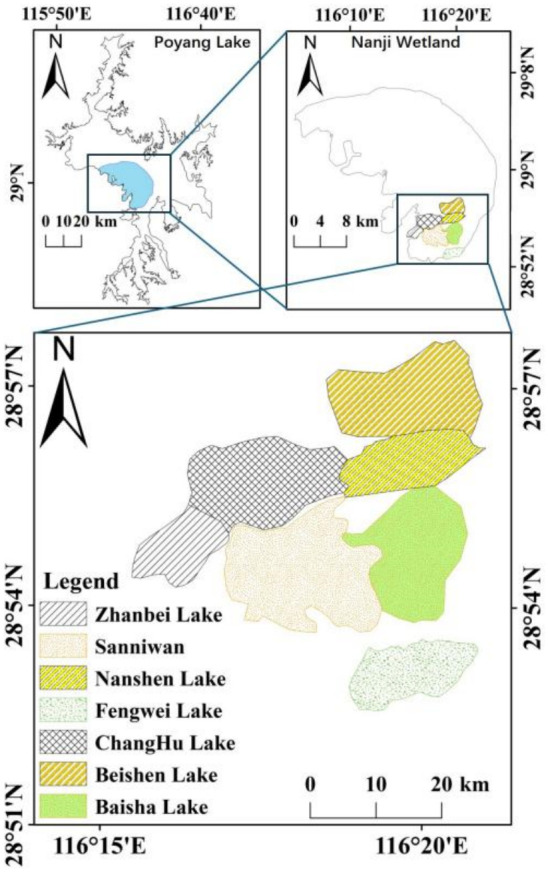
The seven sub-lakes of the Nanji Wetland National Nature Reserve.

The Nanji Wetland National Nature Reserve is located in the southern part of the main basin of Poyang Lake. It was formed by alluvial deposits [37,38]. Seven sub-lakes were selected in the Nanji Wetland of Poyang Lake—Baisha Lake, Beishen Lake, Changhu Lake, Fengwei Lake, Nanshen Lake, Sanniwan, and Zhanbei Lake—to investigate the dynamic distribution of the Black-tailed Godwit and its correlation with landscape patterns (Figure 2).

## 3. Research Methods

### 3.1. Survey of Population Size and Distribution for the Black-Tailed Godwit During the Non-Breeding Season

#### 3.1.1. Collection of Non-Breeding Site Data for the Black-Tailed Godwit

This study collected a total of 11,212 unclassified initial site data for the Black-tailed Godwit from 2011 to 2025 through the China Bird Records Center (http://www.birdreport.cn/, accessed on 15 December 2025) and the Global Biodiversity Information Facility (http://www.gbif.org, accessed on 20 December 2025). First, we excluded breeding site data (stable distribution site data in traditional breeding grounds from May to July each year) from the initial dataset. The remaining site data during the non-breeding season (approximately from August each year to April of the following year) were then imported into ArcGIS 10.8 (Esri, CA, USA), where a 1 km radius circular buffer was created to eliminate site data that were too close together, ensuring that only one site data existed within each buffer. This process resulted in a total of 405 valid site data (including 185 wintering site data in the middle and lower reaches of the Yangtze River) during the non-breeding season, which were saved as CSV files for subsequent analyses [4,26,39].

#### 3.1.2. Survey on Population Dynamics of the Black-Tailed Godwit

This study was conducted from September 2023 to December 2024 using a monocular (SWAROVSKI, 20–60×), employing the direct count method in seven sub-lakes (Baisha Lake, Beishen Lake, Changhu Lake, Fengwei Lake, Nanshen Lake, Sanniwan, and Zhanbei Lake) within the Nanji Wetland National Nature Reserve to investigate population dynamics of the Black-tailed Godwit (Figure 2). Surveys are conducted 1–2 times per month to count the number of Black-tailed Godwits within a 1 km radius. Additionally, in this study, datasets from sub-lakes where the observed Black-tailed Godwit population size exceeded 50 individuals were classified as the high-abundance group, while datasets from sub-lakes with population size below 50 individuals were classified as the low-abundance group. Low-abundance and high-abundance groups were combined into a comprehension group.

### 3.2. Modeling Methodology

#### 3.2.1. Collection and Processing of Environmental Data

A total of 29 environmental variables were selected for this study to simulate potential suitable habitats for the Black-tailed Godwit during the non-breeding season. These 29 variables include climatic factors (bio1-bio19), topographic factors (elevation, slope, aspect, distance to water sources, and distance to beach), human activities (distance to village, distance to paddy field, and distance to road), as well as land use classification and normalized vegetation index (NDVI). Land use classification data were obtained from the “Figshare” platform (https://figshare.com/, accessed on 1 December 2025) at a 30 m resolution [7]. The land use classification data were then imported into ArcGIS software. Using the Extract by Attribute tool, land use types such as water sources, mudflats, paddy fields, and villages were extracted. Subsequently, the Euclidean Distance tool was used to calculate distances to water sources, mudflats, paddy fields, roads, and villages [5,40]. The 19 bioclimatic variables were derived from the 1970–2000 dataset of the Worldlim database (http://www.worldclim.org/, accessed on 2 December 2025) [26,39]. Elevation data were obtained from the elevation dataset on the Geospatial Data Cloud Platform (http://www.gscloud.org/, accessed on 1 December 2025) [40]; the normalized vegetation index was downloaded from the Vegetation Index dataset at the Scientific Information Center for Resources and Environment (https://www.resdc.cn/, accessed on 5 December 2025) [4]. Finally, in ArcGIS, the 29 variables were unified into a single coordinate system (WGS_1984) and resolution (30′, approximately 1 km), masked with the same boundaries, and converted to ASCII file format [26,39,40].

To reduce autocorrelation among environmental variables and improve the predictive accuracy of the model, this study initially imported 29 environmental variables into the MaxEnt model. A preliminary simulation was conducted in combination with occurrence points of the Black-tailed Godwit during the non-breeding season to calculate the contribution rate of each variable to the species’ potentially suitable habitat [1,7]. Subsequently, the extraction tool in ArcGIS was used to obtain the corresponding environmental variable values for each occurrence point. The resulting data were then imported into SPSS 26 software for correlation analysis among the environmental variables [26]. In the Spearman correlation analysis, when the correlation coefficient (*r*) between two variables was ≥|0.8|, the variable with the lower contribution rate was excluded, while the variable with the higher contribution rate was retained [26]. Ultimately, 16 environmental variables were retained for simulating the potential suitable habitat of the Black-tailed Godwit during the non-breeding season (Appendix A Table A1).

#### 3.2.2. Model Parameter Settings and Accuracy Assessment

In this study, the 16 selected environmental variables for the Black-tailed Godwit and its distribution site data during the non-breeding were imported into the model. The model was configured to randomly allocate 75% of the site data for potentially suitable habitat prediction, while the remaining 25% of the site data were used for model accuracy assessment (Appendix A Table A1) [1,26]. Additionally, the Jackknife method and response curve plotting tools were selected in the model configuration interface. The model was then run 10 times, and the average of these 10 runs was used as the final prediction result [1,26]. The model’s predictive accuracy was assessed using the area under the curve (AUC) of the receiver operating characteristic (ROC) curve. Model accuracy is typically classified into the following categories: poor (AUC ≤ 0.6), fair (0.6 < AUC ≤ 0.7), moderate (0.7 < AUC ≤ 0.8), good (0.8 < AUC ≤ 0.9), and excellent (0.9 < AUC ≤ 1) [1,26].

#### 3.2.3. Screening of Dominant Environmental Factors and Delineation of Suitable Habitats

Dominant environmental variables are primarily identified based on their contribution levels. The magnitude of these contribution levels indicates the extent to which each environmental variable influences the distribution of the Black-tailed Godwit during the non-breeding season; environmental variables with high contribution levels are considered the dominant factors influencing the distribution of the Black-tailed Godwit [1,7,26]. Substitution importance can also be used to assess the influence of environmental variables. The model achieves this by randomly rearranging different environmental variables and observing changes in the model’s predictive results. If the model’s results change significantly when the order of a particular environmental variable is altered, the substitution importance of that variable is high [1,7]. The jackknife test method can also evaluate dominant environmental variables using two approaches: “only this variable” and “excluding this variable” [26]. “Only this variable” indicates the effect of a single variable on the model’s results, while “excluding this variable” refers to the model’s prediction after excluding a specific variable. If the model’s results change drastically after excluding a variable, it indicates that the variable has a significant influence [26]. The model’s predicted values range from (0–1), with higher values indicating greater suitability for the species’ survival [7]. In this study, the natural breakpoint method was used to reclassify the suitability values into four habitat categories based on their magnitude: unsuitable (0 ≤ *X* < 0.1), low-suitability habitats (0.1 ≤ *X* < 0.2), mid-suitability habitats (0.2 ≤ *X* < 0.5), and high-suitability habitats (0.5 ≤ *X* ≤ 1) [7,26].

### 3.3. Landscape Index Analysis

#### 3.3.1. Model Data Processing

The study imported the ASC result files from the model’s ten-fold averaged predictions into ArcGIS. Using the natural breakpoint method in the Reclassification tool, the results were divided into four categories. Then, using the Extract by Feature tool in the Spatial Analysis toolset, suitable habitats were extracted into three categories—high, middle, and low—and exported as separate TIF files for subsequent research [41]. The saved high, middle, and low suitability habitat files were imported into ArcGIS as base map data. Vector boundary data in SHP format for three inland lakes—Nanji Wetland, Dongting Lake, and Shengjin Lake—as well as for Nanhuidongtan, Lianyungang Wetland, Yancheng Wetland, as mask data. Finally, using the “Extract by Mask” tool in the Spatial Analysis toolbox, the high-, middle-, and low-suitability habitats for the six regions were extracted and exported separately in TIF format for subsequent analyses.

#### 3.3.2. Satellite Image Acquisition and Supervised Classification

The satellite remote sensing imagery for the Nanji Wetland in this study was obtained from the Landsat 8–9 dataset of the USGS (http://earthexplorer.usgs.gov/, accessed on 7 October 2025). Six remote sensing images with cloud cover less than 5% and a resolution of 30 m, acquired in October 2023 and September–December 2024, were selected. Satellite image data processing was performed using ENVI 5.6.2 software. The six Nanji Wetland satellite images were imported into the software and processed using tools such as radiometric calibration and atmospheric correction [42]. Subsequently, vector boundary data for the seven sub-lakes were imported. The “Crop by Vector Boundary” tool was used to crop the satellite imagery for the corresponding months for each of the seven sub-lakes. Finally, supervised classification of the satellite imagery was performed using the Maximum Likelihood method, categorizing land cover into four classes: deep water (SSQ), shallow water (QSQ), mudflats (ND), and vegetated areas (ZB). The results were then exported in TIF format using ArcGIS 10.8 software for subsequent analyses.

#### 3.3.3. Landscape Analysis

The prepared TIF files for the study area were imported into Fragstats 4.2 software to calculate landscape indices at the landscape scale. When calculating landscape indices for each sub-lake in the Nanji Wetland, the following parameters were selected in the software: number of patches (NP), patch density (PD), Maximum Patch Index (LPI), Division Index (DIVISION), Split Index (SPLIT), Aggregation Index (AI), Shannon Diversity Index (SHDI), and Shannon Evenness Index (SHEI). When calculating landscape indices for the middle and lower reaches of the Yangtze River, six landscape pattern indices were selected: NP, PD, LPI, DIVISION, SPLIT, and AI. The DIVISION and SPLIT reflect landscape fragmentation; higher values indicate more severe fragmentation [10,37]. The SHDI reflects landscape richness, while the Shannon Evenness Index reflects landscape fragmentation. A higher SHDI value indicates greater diversity of land use types and a higher degree of fragmentation [8,9,10,12]. The SHEI ranges from 0 to 1; values closer to 1 indicate a higher degree of landscape fragmentation [37]. The AI represents the connectivity of patches within the landscape pattern; higher values indicate denser habitat patches and higher connectivity, while lower values indicate severe habitat fragmentation and lower connectivity [9,10].

### 3.4. Correlation Between the Wintering Distribution Patterns of the Black-Tailed Godwit and Landscape Indices

This study imported data on the population size of the Black-tailed Godwit in seven sub-lakes of the Nanji Wetland (18 October 2023; September 9th, October 11th, November 12th and 24 November 2024) and the corresponding landscape pattern data for the seven sub-lakes—including indicators related to different land-use areas—into SPSS. We employed Spearman’s test to analyze the correlations among population size, landscape indices, and land-use areas. Differences were considered statistically significant at a *p*-value less than 0.05. We have categorized the correlations in the text as weak (|*r*| < 0.3), moderate (0.3 ≤ |*r*| < 0.7), and strong (|*r*| ≥ 0.7) [8]. Finally, the results were imported into Origin 2024b software to generate a correlation heatmap.

### 3.5. Significance Analysis of Landscape Indices

In this study, landscape index data for the three coastal wetlands and three inland wetlands, as well as landscape index and land use area data for lakes corresponding to the high-abundance and low-abundance groups of Black-tailed Godwits, were imported into SPSS. An independent samples *t*-test was used to assess the significance of differences among them.

## 4. Results

### 4.1. Model Results for the Distribution of the Black-Tailed Godwit

#### 4.1.1. Model Accuracy

**Figure 3:**
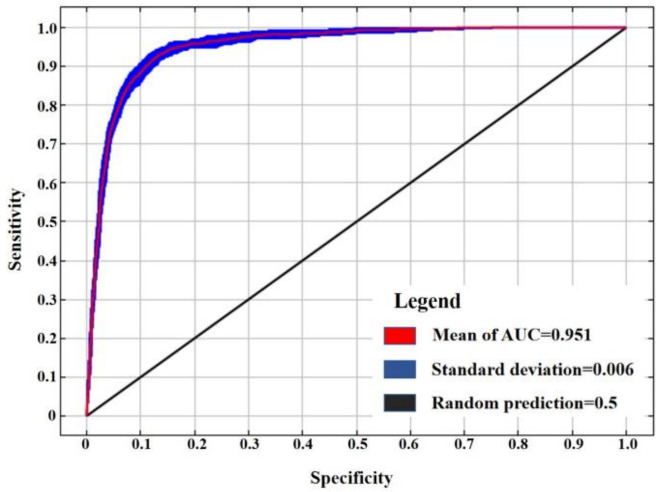
ROC curve for model accuracy.

The AUC value for the model’s 10 runs average results was 0.951, with a standard deviation of 0.006. These results demonstrate that the model developed in this study provides accurate and reliable predictions for the distribution of the Black-tailed Godwit during the non-breeding season (Figure 3).

#### 4.1.2. Dominant Environmental Variables and Response Curves

**Figure A1:**
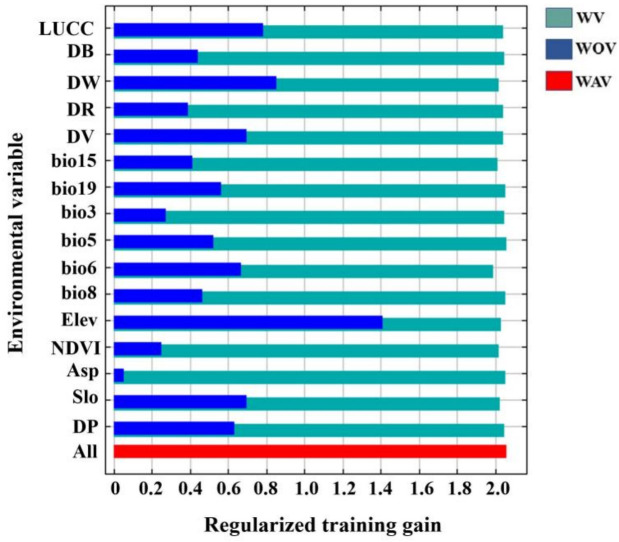
The jackknife test result of the environmental variables. (Note: WV represents without the variable, WOV represents with only variable, and WAV represents with all variables).

Based on the results of 10 modeling runs and the jackknife method, the dominant environmental factors influencing habitat suitability for the Black-tailed Godwit during the non-breeding season are elevation, distance to water source, slope, distance to paddy field, land use classification, and minimum temperature of the coldest quarter (Appendix A Table A2, Figure A1).

**Figure 4:**
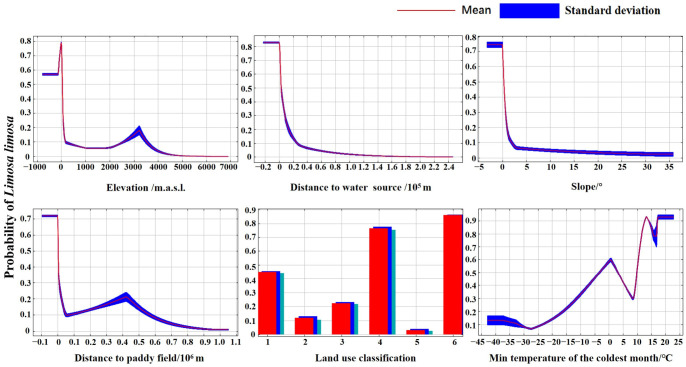
Response curves for dominant environmental variables. (Note: Land use classification were coded as follows: 1 represents cropland, 2 represents forest land, 3 represents grassland, 4 represents water bodies, 5 represents urban areas, and 6 represents unutilized land).

The Black-tailed Godwit is most influenced by elevation, preferring to forage in areas below 36.16 m.a.s.l. It prefers to forage within 2.87 km of water sources; areas with a slope greater than 0.26 are unsuitable for its activities. The Black-tailed Godwit prefers to forage within 1.91 km of paddy fields and prefers land use types such as water bodies, mudflats, and marshes; the minimum temperature of the coldest quarter has a certain influence on the Black-tailed Godwit distribution, with areas where temperatures range from −3.35 to 3.14 °C and those exceeding 9.90 °C being suitable for its activities (Figure 4).

#### 4.1.3. Potentially Suitable Habitats

**Figure 5:**
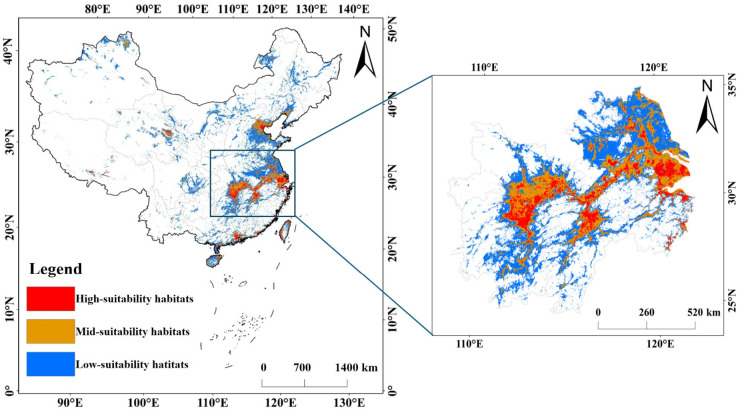
Potential suitable habitats for the Black-tailed Godwit during the non-breeding season.

The area of high-suitability habitats for the Black-tailed Godwit during the non-breeding season is 128,800 km^2^, primarily distributed in the middle and lower reaches of the Yangtze River basin, such as Dongting Lake, Liuye Lake, Maoli Lake, and Datong Lake in Hunan; Honghu Lake, Huanggai Lake, Houguan Lake, Chenhu Lake, Zhangdu Lake, Futou Lake and Liangzi Lake in Hubei; Poyang Lake, Aixi Lake, Jinxi Lake and Junshan Lake in Jiangxi; Daguan Lake, Shengjin Lake, Caizi Lake and Chaohu Lake in Anhui; Taihu Lake, Gaoyou Lake, Hongze Lake, Shijiu Lake, Changdang Lake and coastal wetlands in Jiangsu; certain reservoirs and coastal wetlands in Zhejiang; and coastal wetlands in Shanghai. The area of the mid-suitability habitat is 40,310 km^2^, primarily distributed around the highly suitable zones; the area of the low-suitability habitat is 123,270 km^2^, and most of these low-suitability zones are located around the highly and moderately suitable zones in eastern and southern China (Figure 5).

### 4.2. Differences in Landscape Indices

In the high-suitability habitats for the Black-tailed Godwit, landscape fragmentation indices (NP, PD, DIVISION, SPLIT) and connectivity indices (AI) were both lower and higher in the high-suitability habitats than those in the mid- or low-suitability habitats, respectively (Appendix A Table A3). The high-suitability habitats had low numbers of patches, division indices, and split indices, as well as low patch density; conversely, they had high aggregation indices (Appendix A Table A3).

In the high-suitability habitats of the three inland lakes (Shengjin Lake, Dongting Lake, and Nanji Wetland), the NP (2.33 ± 0.58), PD (0.01 ± 0.004), DIVISION (0.01 ± 0.01), and SPLIT (1.01 ± 0.01) were all lower than those in the high-suitability habitats of the three coastal regions. However, the AI (90.73 ± 1.81) and LPI (99.42 ± 0.49) were both higher than those of the three coastal wetlands (AI: 59.61 ± 19.02; LPI: 56.11 ± 31.43) (Table 1).

### 4.3. Correlation Between the Distribution and Landscape Pattern of the Black-Tailed Godwit in the Nanji Wetland

**Figure 6:**
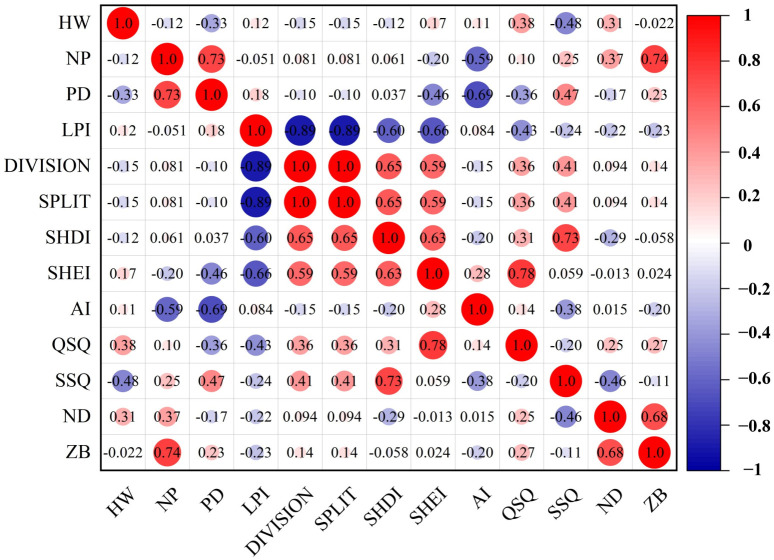
Correlation analysis of Black-tailed Godwit population distribution and landscape indices (Note: HW represents the Black-tailed Godwit population. The same below).

**Figure A2:**
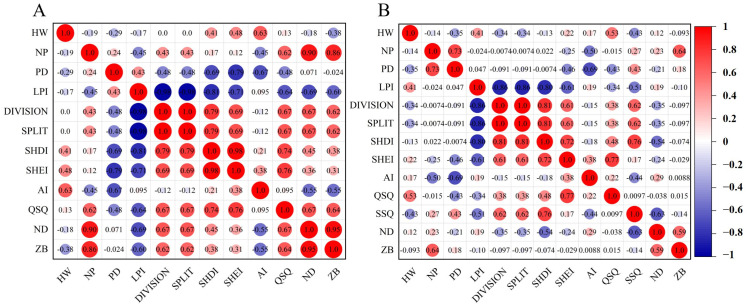
Correlation analysis of high- and low-abundance Black-tailed Godwit groups with landscape indices. (Note: (**A**) represents the high-abundance group, (**B**) represents the low-abundance group).

The distribution of the Black-tailed Godwit in Nanji Wetland showed a significant moderate positive correlation with shallow-water area (*r* = 0.38, *p* < 0.05) and a significant moderate negative correlation with deep-water area (*r* = −0.48, *p* < 0.01) (Figure 6). The high-abundance group of Black-tailed Godwits did not exhibit statistically significant correlations with any landscape indices, whereas the low-abundance group showed a significant moderate positive correlation with shallow-water area (*r* = 0.53, *p* < 0.05) (Appendix A Figure A2).

A *t*-test revealed that there were no significant differences in landscape indices or habitat area between the high-abundance and low-abundance groups of Black-tailed Godwits in the Nanji Wetland. However, the high-abundance group exhibited higher values for five indicators—NP, LPI, QSQ, ND, and ZB—compared to the low-abundance group, while other indicators were lower in the high-abundance group (Appendix A Table A4).

## 5. Discussion

### 5.1. Model Results and Dominant Environmental Variables

The average training AUC across ten runs was 0.9616, and the average testing AUC was 0.9515, resulting in a difference of less than 0.05, indicating no overfitting in this model. This result demonstrates that the predictions from this study align with the Black-tailed Godwit’s habitat selection requirements during the non-breeding season and can accurately identify its potential suitable habitats.

Altitude constitutes a critical factor influencing avian distribution, as the cold, hypoxic conditions of high-altitude regions elevate birds’ energy expenditure [7,43,44]. Low-altitude regions, characterized by extensive water systems and abundant food resources, serve as preferred foraging grounds for most waterbird species [45,46]. Consequently, the Black-tailed Godwit predominantly inhabits low-altitude areas (below 36.16 m.a.s.l.), a pattern similar to that observed in the Siberian Crane (*Leucogeranus leucogeranus*) (below 22 m.a.s.l.) and the Hooded Crane (*Grus monacha*) (below 22.3 m.a.s.l.) [7,26]. However, relative to the Black-tailed Godwit, the Spotted Redshank (*Tringa erythropus*) and Common Crane (*Grus grus*) demonstrate greater environmental tolerance and are capable of occupying higher-altitude habitats [3,39]. The Black-tailed Godwit prefers to forage in areas with gentle slopes, as these regions offer flat terrain and abundant water sources, providing ample food resources [19,21,22]. This study found that during the non-breeding season, the Black-tailed Godwit shows a stronger preference for land types such as water bodies, mudflats, and marshes, and are active in areas located within 2.87 km of water sources and 1.91 km of paddy fields. Consequently, the Black-tailed Godwit exhibits a high degree of dependence on water sources. Temperature is the primary factor limiting the northernmost extent of avian wintering ranges; in regions with lower minimum temperatures during the coldest month, water bodies freeze to varying degrees during the non-breeding season [7,47]. The Black-tailed Godwit primarily forages in water bodies; ice cover hinders its foraging and simultaneously increases its energy expenditure [47]. Therefore, areas with relatively higher minimum temperatures in the coldest month can provide suitable foraging grounds for the Black-tailed Godwit.

### 5.2. Response of the Distribution of the Black-Tailed Godwit to Landscape Pattern

Habitats exhibiting high fragmentation and low connectivity are unable to support substantial waterbird populations [8]. In this study, high-suitability habitats for the Black-tailed Godwit displayed higher aggregation index (AI), whereas NP, DIVISION, SPLIT, and PD were lower. These findings suggest that high-suitability habitats exhibit low fragmentation and high connectivity, thereby providing high-quality environments for the Black-tailed Godwit. Areas with elevated hydrological connectivity generally support higher densities of benthic organisms; thus, hydrological connectivity may indirectly enhance the Black-tailed Godwit’s foraging efficiency by influencing benthic organism abundance [48,49,50].

The study found that in high-suitability habitats of the three inland lakes, PD, DIVISION, and SPLIT were lower than in coastal wetlands, while AI and LPI were comparatively higher. These observations indicate that inland lakes constitute wetlands characterized by low fragmentation and high connectivity. The principal factors affecting the ecological stability of lake and coastal wetland ecosystems are water resources and hydrological connectivity, and climate change, respectively [35]. Climate change induces sea-level rise and modifies wetland biogeochemical cycles and plant community structures; consequently, it is regarded as a major threat to wetland ecosystems [35,51,52,53]. Coastal wetland reclamation has resulted in reduced wetland area and increased fragmentation. Furthermore, human activities have intensified alarm responses in the Black-tailed Godwit, ultimately contributing to the fragmentation and reduction of its natural habitat [23,27,28,35]. Recent surveys of waterbird diversity reveal that the non-breeding population of the Black-tailed Godwit in coastal wetlands across multiple regions is gradually declining; in certain areas, it no longer remains the dominant species [23,27,28]. Consequently, during the non-breeding season, the Black-tailed Godwit is expanding its range into inland areas characterized by well-developed water systems, high connectivity, and low fragmentation.

### 5.3. Correlation Between the Distribution of the Black-Tailed Godwit and Landscape Indices

Shorebirds prefer to forage for aquatic invertebrates in mudflats and open shallow-water areas, where their food resources typically inhabit the 0–6 cm sediment layer of mudflats and shallow-water areas [21,22,37,54]. Previous studies indicate that Nanji Wetland is primarily composed of islands and lakes [8,54]. Most shorebirds in Nanji Wetland are concentrated in three lakes (Changhu Lake, Sanniwan, and Beishen Lake) and their distribution area necessarily includes three landscape elements: water bodies, vegetation, and bare land [54]. This study found the distribution of Black-tailed Godwits in Nanji Wetland exhibits a significant moderate positive correlation with shallow-water areas. Conversely, a significant moderate negative correlation was observed with deep-water areas (Figure 6). Black-tailed Godwits possess relatively long bills and legs, enabling them to forage in shallow-water areas and mudflats with a certain water level, where food resources are also abundant [54]. In deep-water areas where the water level is too high, Black-tailed Godwits cannot wade in, nor can their bills penetrate the sediment layer for foraging. Furthermore, an excessive extent of deep-water areas reduces the area of suitable habitats such as shallow-water areas and mudflats. During field surveys, our research team observed that Black-tailed Godwits preferentially forage in unplanted paddy fields during the non-breeding season. However, once post-planting and vegetation cover becomes excessively dense, the Black-tailed Godwits will leave these areas. This phenomenon indicates that Black-tailed Godwits prefer areas with low vegetation cover, because excessive vegetation cover reduces foraging efficiency and also compresses the area of shallow-water areas and mudflats [21]. Therefore, the effects of vegetation cover on distribution of Black-tailed Godwits require further research.

Landscape heterogeneity is also a primary factor influencing habitat selection by shorebirds [54,55]. However, no significant correlations were found in this study between the distribution of Black-tailed Godwits in Nanji Wetland and changes in landscape indices. This may be due to the relatively small size of our study area and the close proximity of sub-lakes in Nanji Wetland, which lead to little variation in landscape indices. Nevertheless, this study suggests that landscape fragmentation exerts a certain influence on the distribution of Black-tailed Godwits, with larger numbers distributed in sub-lakes exhibiting high habitat connectivity. A larger maximum patch area indicates lower habitat fragmentation [37]. A higher aggregation index represents greater habitat connectivity [9,10]. The results show that Black-tailed Godwits in Nanji Wetland prefer lakes with high habitat aggregation indices. Studies have shown that areas with high connectivity in inland wetlands exhibit higher benthic macroinvertebrate richness [35,56,57]; consequently, such areas can enhance the foraging efficiency of Black-tailed Godwits. In areas with high fragmentation, the food resources for Black-tailed Godwits are adversely affected, and anthropogenic activities are relatively elevated. As a result, Black-tailed Godwits experience increased vigilance and reduced foraging efficiency, leading to elevated energy expenditure [57].

## 6. Conclusions

This study employed the MaxEnt model and landscape indices to identify high-suitability habitats for the Black-tailed Godwit during the non-breeding season, primarily located in the middle and lower reaches of the Yangtze River, and to determine elevation, distance to water sources, slope, distance to paddy field, land use type, and minimum temperature of the coldest quarter as dominant environmental variables. Landscape fragmentation, connectivity, human disturbance, and climate change were found to be associated with the species’ shift from coastal to inland habitats. Within the sub-lakes of the Nanji Wetland, population size showed significant moderate negative correlation with deep-water areas, and significant moderate positive correlation with shallow-water areas. At the large wetland scale (coastal and inland wetlands), connectivity and fragmentation exerted a stronger influence, whereas at the small scale (Nanji Wetland), shallow-water areas and food resources were more critical. This study is limited by the spatial resolution of the selected environmental variables and the remote sensing data. These findings also can provide a fundamental reference framework for Black-tailed Godwit conservation.

## Figures and Tables

**Table 1:** Differences in landscape indices between coastal and some inland regions.

Area	Landscape Indices (Mean ± SD)					
	NP	PD	LPI	DIVISION	SPLIT	AI
Coastal regions	6.33 ± 3.51	0.23 ± 0.18	56.11 ± 31.43	0.57 ± 0.36	3.65 ± 2.57	59.61 ± 19.02
Inland regions	2.33 ± 0.58	0.01 ± 0.004	99.42 ± 0.49	0.01 ± 0.01	1.01 ± 0.01	90.73 ± 1.81

## Data Availability

The data presented in this study are available request upon from the corresponding author. The data are not publicly available due to the constraint in the consent.

## Appendix A

**Table A1:** Environment variables and their descriptions.

Variable	Description	Unit	Used Variables in the Model
bio 1	Annual mean temperature	°C	
bio 2	Mean diurnal temperature range	°C	
bio 3	Isothermality	-	√
bio 4	Temperature seasonality	-	
bio 5	Max temperature of the warmest month	°C	√
bio 6	Min temperature of the coldest month	°C	√
bio 7	Temperature annual range	°C	
bio 8	Mean temperature of the wettest quarter	°C	√
bio 9	Mean temperature of the driest quarter	°C	
bio 10	Mean temperature of the warmest quarter	°C	
bio 11	Mean temperature of the coldest quarter	°C	
bio 12	Annual precipitation	mm	
bio 13	Precipitation of the wettest month	mm	
bio 14	Precipitation of the driest month	mm	
bio 15	Precipitation seasonality (coefficient of variation)	-	√
bio 16	Precipitation of the wettest quarter	mm	
bio 17	Precipitation of the driest quarter	mm	
bio 18	precipitation of the warmest quarter	mm	
bio 19	Precipitation of the coldest quarter	mm	√
Elev	Elevation	m.a.s.l.	√
NDVI	Normalized vegetation index	-	√
LUCC	Land use classification	-	√
DP	Distance to paddy field	m	√
DW	Distance to water source	m	√
DB	Distance to mudflat	m	√
DR	Distance to road	m	√
Asp	Aspect	-	√
Slo	Slope	°	√
DV	Distance to village	m	√

**Table A2:** Percentage contribution and permutation importance of the environmental variable.

Variable	Percent Contribution/%	Permutation Importance/%
Elevation	29.6	19.8
Distance to Water source	17.8	2.2
Slope	13	9.6
Distance to paddy field	8.9	1.3
Land use classification	7.3	1.8
Min temperature of the coldest month	6.6	32.6

**Table A3:** Regional variability of landscape indices.

Areas	NP	PD	LPI	DIVISION	SPLIT	AI
Nationwide						
High-suitability habitats	3892	0.04	14.36	0.94	17.81	76.75
Mid-suitability habitats	12,803	0.05	12.34	0.96	23.80	72.78
Low-suitability habitats	39,460	0.05	21.10	0.95	18.66	73.12
Nanhuidongtan Wetland						
High-suitability habitats	3	0.03	90.91	0.17	1.20	80.08
Mid-suitability habitats	7	0.12	27.78	0.77	4.32	62.20
Low-suitability habitats	0	0	0	0	0	0
Lianyungang Wetland						
High-suitability habitats	10	0.29	29.79	0.84	6.33	42.50
Mid-suitability habitats	18	0.05	46.57	0.66	2.98	80.86
Low-suitability habitats	9	0.05	90.57	0.18	1.21	85.75
Yancheng Wetland						
High-suitability habitats	6	0.37	47.62	0.71	3.42	56.25
Mid-suitability habitats	31	0.05	63.73	0.57	2.32	77.40
Low-suitability habitats	17	0.01	76.81	0.38	1.62	87.18
Shengjin Lake						
High-suitability habitats	2	0.01	99.45	0.01	1.01	88.72
Mid-suitability habitats	6	0.47	41.67	0.75	4.00	35.29
Low-suitability habitats	9	0.07	32.56	0.77	4.42	65.11
Dongting Lake						
High-suitability habitats	2	0.0019	99.90	0.00	1.00	92.23
Mid-suitability habitats	23	0.20	45.28	0.77	4.31	56.54
Low-suitability habitats	0	0	0	0	0	0
Nanji Wetland						
High-suitability habitats	3	0.01	98.91	0.02	1.02	91.25
Mid-suitability habitats	7	0.21	65.63	0.54	2.20	61.54
Low-suitability habitats	0	0	0	0	0	0

**Table A4:** Landscape indices for the high-abundance group and the low-abundance group.

Groups	Landscape Index (Mean ± SD)												
	HW	NP	PD	LPI	DIVISION	SPLIT	SHDI	SHEI	AI	QSQ	SSQ	ND	ZB
A	382 ± 435	66.4 ± 44	10.9 ± 5.1	41.3 ± 8.7	0.7 ± 0.05	3.5 ± 0.7	1 ± 0.04	0.8 ± 0.1	87.9 ± 1.4	1.5 ± 0.9	0	2.3 ± 1.1	2.4 ± 0.9
B	6.3 ± 10.9	64.3 ± 35.5	10.9 ± 4.7	39.6 ± 6.9	0.7 ± 0.05	3.7 ± 0.7	1.1 ± 0.2	0.8 ± 0.1	88.1 ± 2	1.4 ± 1	0.4 ± 0.8	2 ± 0.9	2.3 ± 0.8

(Note: A represents the high-abundance group; B represents the low-abundance group).
